# Prevalence and Associated Factors of Sharps Injury Amongst Nurses: A Cross-Sectional Study

**DOI:** 10.7759/cureus.96650

**Published:** 2025-11-12

**Authors:** Priyanka Elizabeth Thomas, Rathish Nair, Visanth VS, Girishkumar K

**Affiliations:** 1 Nursing, All India Institute of Medical Sciences, Patna, IND

**Keywords:** biohazards, needle stick injury, nurses, occupational hazards, post-exposure prophylaxis, prevalence, sharps injury, teaching hospital, tertiary care centre

## Abstract

Background: Sharps injuries remain a prevalent occupational hazard for nurses, with implications for both physical and psychological health. Even minor injuries can lead to heightened anxiety due to the potential risk of infection.

Aim: This study aimed to assess the prevalence of sharps injuries and identify associated risk factors among nurses in a tertiary care hospital setting.

Materials and methods: A cross-sectional study was conducted from September to November 2023 among 339 nurses employed at a public sector, tertiary care teaching hospital in Bihar, India. Participants were selected using a convenience sampling method. Data were collected through a structured, self-administered questionnaire distributed via a Google Form. Both descriptive and inferential statistical analyses were employed.

Results: The prevalence of sharps injuries among participants was 8.84%. Injuries were more frequently reported in high-demand units and during tasks involving the preparation or use of medical devices.

Conclusion: The overall prevalence of sharps injuries was relatively low. However, the findings underscore the importance of targeted safety interventions. Implementing unit-specific precautions and enhancing biomedical waste management practices are essential to improving occupational safety and reducing injury risks among nurses.

## Introduction

Health care personnel are at high risk of exposing themselves to sharp injuries in work settings daily. These risks are higher due to the increased number of invasive medical procedures performed in a health care setting [1]. Despite improvements in infection control, injuries from needle sticks, sharp objects, and blood and bodily fluid exposure at work still occur; are considered to be serious concerns for public health. According to a World Health Organization (WHO) report released in Geneva, over two million sharps injuries occur at work every year for around 35 million healthcare workers [2]. These injuries could have a greater impact on the physical and psychological health, which may affect the overall work performance of an employee. Sharps injury is considered a major hazard among health care personnel in their work settings, resulting from wounds caused by accidental puncturing of skin by sharps [2].

Items used for patient care services include razor blades, scalpels, lancets, scissors, clamps, retractors, staples, glass objects, intravenous cannulas, and needles, including hypodermic and blood collection needles [3]. Invasive procedures such as surgeries, suturing, needle recapping, or disposal and collection of blood could result in an injury with sharps. According to the Centers for Disease Control and Prevention (CDC) guidelines from Atlanta, based on surveillance data collected in the United States between 1981 and 2020, sharps injuries are largely preventable but may result in healthcare workers acquiring up to 20 different blood-borne pathogens [4]. Each year, an estimated 385,000 needlestick and other sharps-related injuries occur among hospital-based healthcare personnel in the United States [4].

According to a 2003 study by Prüss-Ustün et al., 200-5000 HIV infections, 66,000 hepatitis B infections, and 16,000 hepatitis C infections are caused by sharps injuries [5]. Another study conducted by Akyol and Kargin in 2016 reported that the common excuses for failing to disclose injuries included “too busy to report it” and the “source was non-infectious” [6]. The burden of sharps injury to those exposed could be financial, emotional, and social, with the latter including the loss of professional experience and associated litigation.

Some aspects of hospital environment might contribute to incidents with sharps, such as work intensification, performing simultaneous activities, and frequent interruptions during task performance [7]. Additionally, nurses were more prone to get injuries from needle sticks or sharp objects while on the job if they lacked understanding or had not received training on workplace preventive techniques. [8] To ascertain the prevalence of needle stick injuries (NSIs) among nurses, Laishram et al. (2013) carried out a cross-sectional study in a tertiary hospital in Imphal [8]. With a stated incidence of 28.1%, intravenous injections are the most common action that causes injury [8]. Remarkably, 43% failed to report the injuries, and 96% did not wear gloves when exposed to the threat. Although there was widespread awareness of post-exposure prophylaxis, its actual use was not very high. The study underlined the need to promote safety precautions and reporting [9].

Prados et al. (2025) investigated the relationship between 348 nurses in Ecuador who worked shifts, experienced sleep problems, and experienced NSIs [10]. Particularly at risk were female nurses and less experienced nurses. Strategies for prevention were proposed to reduce occupational injuries [10]. Sharp injuries were the most frequent type of occupational injury, frequently reported among cleaning workers and nurses, according to a study by Medeni et al. (2025) that examined 694 reported occupational injuries at a Turkish university hospital between 2020 and 2023 [11]. Approximately 30% of workers who were hurt did not wear personal protective equipment. Another study emphasized the need for more effective surveillance systems and particular safety procedures that can prevent such accidents among healthcare professionals, but it concentrated mostly on occupational injuries rather than solely sharp injuries [11]. The significance of training among healthcare personnel was demonstrated by the study by Wong et al. (2025), which indicated that rates in NSIs considerably lowered with training programs [12].

A systemic failure in promoting adequate reporting methods is indicated by the high incidence of injuries and the underreporting of injuries. Furthermore, there is frequently a dearth of studies on the main risk factors, which include work overloads, a lack of training in infection prevention, and a lack of preventative education in medical education programs. The frequency of these injuries is rather high, even with several protocols and preventive measures in place. This is frequently made worse by inadequate training, excessive workloads, a lack of commitment to safety protocols, and a failure to report incidents. Being the largest and most frontline members of the healthcare profession, nurses are particularly vulnerable to sharp accidents. They run the risk of suffering a sharps injury while performing routine clinical operations, including blood draws, sutures, and injections. The prevalence and contributing factors have not been thoroughly assessed, despite their high level of risk exposure, particularly in tertiary care settings. This gap needs to be filled to come up with proper prevention strategies, reinforce occupational health policies, and eventually protect the wellness and protection of nursing professionals. Although NSIs have been the subject of much investigation, few studies have looked at nurses' exposure to other sharp objects in the workplace. Thus, this research aims to determine the frequency of sharps injuries and to pinpoint risk variables among nurses working in a tertiary care facility in Bihar, India.

## Materials and methods

**Figure 1:**
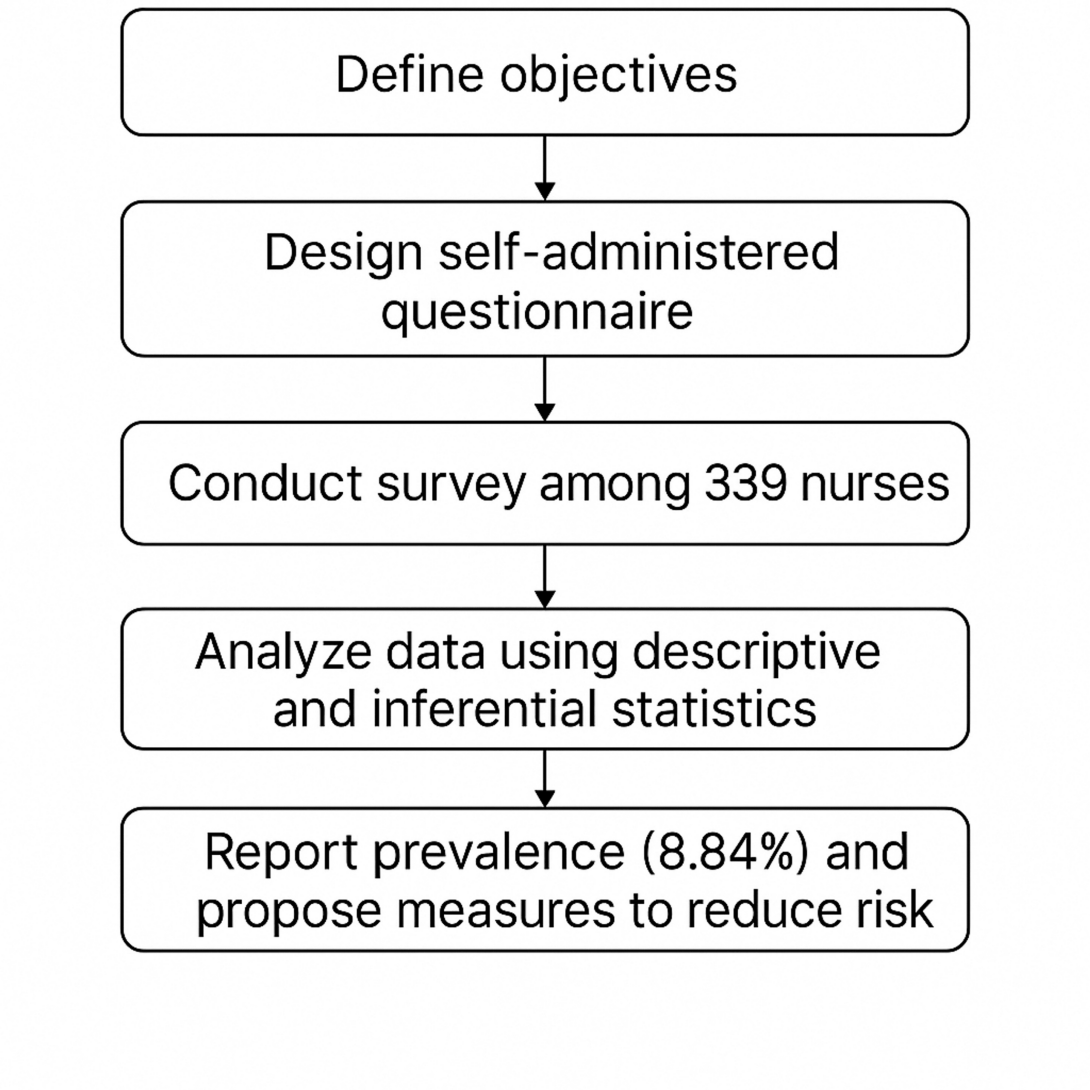
Workflow of the study on prevalence and associated factors of sharps injury among nurses

Figure 1 shows a workflow diagram outlining the stepwise approach followed in this cross-sectional study. Initially, clear objectives were defined to guide the research direction. To get pertinent information from the participants, a self-administered questionnaire (see Appendices) was created. The Google Forms (Google LLC, Mountain View, CA, USA) questionnaire was used to administer a survey to 339 nurses.

Study design and setting

A single-centric cross-sectional study was performed among nurses between September and November 2023 in a 1000-bedded multispecialty, tertiary care, public sector hospital in Bihar state, India.

Sub-objectives

The purpose of this study was to determine the occurrence of sharps injuries and look into risk variables for these injuries among nurses employed by a teaching hospital that provides tertiary care.

Sample size and sampling technique

Several common techniques were used to determine the sample size: Taro Yamane formula (n = 281 for a population of 941 nurses), Raosoft sample size calculator (Raosoft Inc., Seattle, WA) (n = 273 for a population of 941 nurses), and the Krejcie and Morgan table (n = 269 for a population of 900 nurses), with ±5% margin of error at 95% confidence interval [13,14,15]. A total of 339 responses were obtained, and accounting for 10% contingency, it was more than the minimum required sample size, which met the statistical requirements. A convenience sampling method was used to choose respondents because of the variety in work area and duty program among nursing professionals (N=941) in the research setting. Random sampling method was not utilized because all the nurses were dispersed in various work areas and at different work schedules, and they were not easily accessible in one sitting for sampling.

Ethical considerations

Participants in the study were made aware of its objectives, and after receiving institutional ethics committee permission (Ref. No. AIIMS/Pat/IRC/2022/999), informed consent was acquired. Confidentiality, autonomy, and anonymity were guaranteed to study participants.

Validation and pilot testing

Content validity of the questionnaire was determined through consultation with three experts in the domain of the questionnaire, with a Content Validity Index (CVI) of 0.80. Expert recommendations were used to improve the tool. Pilot testing was done with 30 nurses (10% of the minimum required sample size) to test feasibility, and these nurses were excluded from the main study after pilot testing. Cronbach's alpha was employed to evaluate internal consistency reliability; a result above 0.70 is considered satisfactory.

Inclusion and exclusion criteria

Inclusion Criteria

Nurses who directly care for patients in inpatient and outpatient departments, and those who agreed to participate.

Exclusion Criteria

Nurses in supervisory or administrative roles, and those nurses involved in direct patient care in inpatient and outpatient departments, who were unwilling to participate.

Data collection tool

Based on prior research and expert advice, a structured, self-administered questionnaire in English (see Appendices) was created. In order to ascertain the frequency and contributing variables of sharps injuries among nurses, the final version contained both multiple-choice and categorical items.

Section 1

Baseline characteristics, including socio-demographic and professional information (age, gender, marital status, education, professional qualification, unit of work, work experience, Hepatitis B vaccination status, training received on biomedical waste and post-exposure prophylaxis).

Section 2

Details regarding prevalence and associated factors, such as the frequency of injuries from sharp objects, the kind of device that caused the injury, the activity being engaged in at the time of the accident, the location of the damage, and the extent of the injury.

Data collection procedure

All nurses were given the right to participate or not. Moreover, no contact was made between researchers and participants to avoid any coercion. The first page of the Google Form survey had information on informed consent, and by opting to provide consent, they would be redirected to fill out the Google Forms questionnaire. Of the 941 working nurses in the hospital, 339 nurses were included in the research after meeting the inclusion criteria. A huge (N=941 nurses) number of working nurses in varied units were invited to take part in the research, so samples were chosen using the convenience sampling technique. An online survey with data collection instruments consisting of informed consent and a questionnaire was created using Google Forms and disseminated to nurses working in the institute. The respondents were instructed to orient themselves with the study title, purpose, and objectives, and if they agreed, the Google Form was filled out and submitted.

Data management and statistical analysis

IBM SPSS Statistics for Windows, version 25 (IBM Corp., Armonk, NY, USA) was utilized for the analysis after the data from the Google Forms was exported to Microsoft Excel (Version 2017) (Microsoft Corp., Redmond, WA, USA). Baseline variables and exposure rates were summarized using descriptive statistics (frequency, percentage). The Chi-square and Fisher's exact tests were used as inferential statistics to determine whether baseline characteristics and sharps injuries were related, while keeping the significance level suitable for cross-sectional research. Multivariate analysis was performed to identify independent predictors and the possibility of collinearity between variables.

## Results

Baseline characteristics of respondents

Of the total, 227 (67%) were female respondents. Two hundred and seventeen (64%) of participants were between the ages of 25-29 years. More than half (180, 53.1%) of the participants were unmarried. The majority of research participants, 261 (77%), were B.Sc nurses. One to five years of work experience is possessed by 189 nurses (57.2%). Regarding workplace distribution, the majority of participants worked in wards 130 (38.3%), followed by intensive care units 74 (21.8%), outpatient departments 37 (14.7%), emergency rooms 26 (7.7%), and labour rooms 17 (5.0%). In terms of hepatitis B immunization status, 172 (50.7%) were completely vaccinated, 86 (25.4%) were partially vaccinated, and 81 (23.9%) were unvaccinated. A large proportion of participants had received at least one training session on biomedical waste management 269, 79.4%) and post-exposure prophylaxis 227 (67%) after joining the institute (Table 1).

**Table 1:** Baseline characteristics of respondents working at a tertiary care center, Bihar (n=339)

Variables	Frequency	Percentage (%)
Age		
20-24	29	8.6
25-29	217	64.0
30-34	84	24.8
35-39	9	2.7
Gender		
Male	112	33
Female	227	67
Marital status		
Unmarried	180	53.1
Married	159	46.9
Educational status		
General nursing and midwifery	36	10.6
Post B.Sc Nursing	26	7.7
B.Sc Nursing	261	77.0
M.Sc Nursing	16	4.7
Working unit		
Emergency room	26	7.7
OPD	50	14.7
Wards	130	38.3
Intensive care unit (ICU)	74	21.8
Operating room	37	10.9
Labour room	17	5.0
Blood bank	2	0.6
Vaccination area	3	0.9
Years of experience		
Less than 1 year	93	27.4
1-5 years	194	57.2
6-10 years	48	14.2
11-15	3	0.9
More than 15 years	1	0.3
Hepatitis B vaccination status		
Completely vaccinated	172	50.7
Partially vaccinated	86	25.4
unvaccinated	81	23.9
Training on biomedical waste		
Received	269	79.4
Not received	70	20.6
Training on post-exposure prophylaxis		
Received	227	67
Not received	112	33

Prevalence of sharps injury

**Figure 2:**
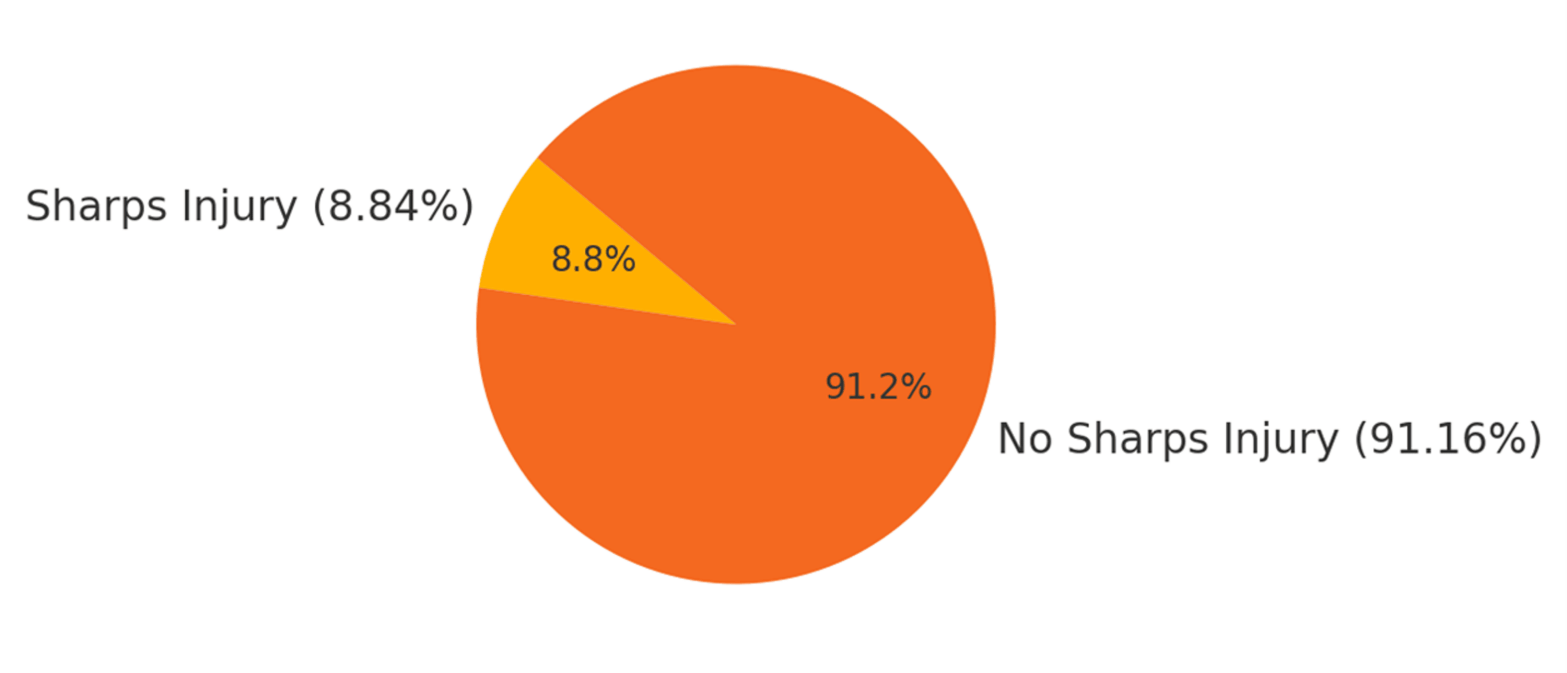
Prevalence of sharps injury among nurses

The study included 339 nurses in total. Thirty of these nurses reported being injured by sharp objects, yielding an 8.84% prevalence rate (Figure 2). Of those injured, 60% had been injured at least once, and 40% had been exposed up to five times in the previous 12 months (Table 2).

Occupational exposure to sharps injury

**Figure 3:**
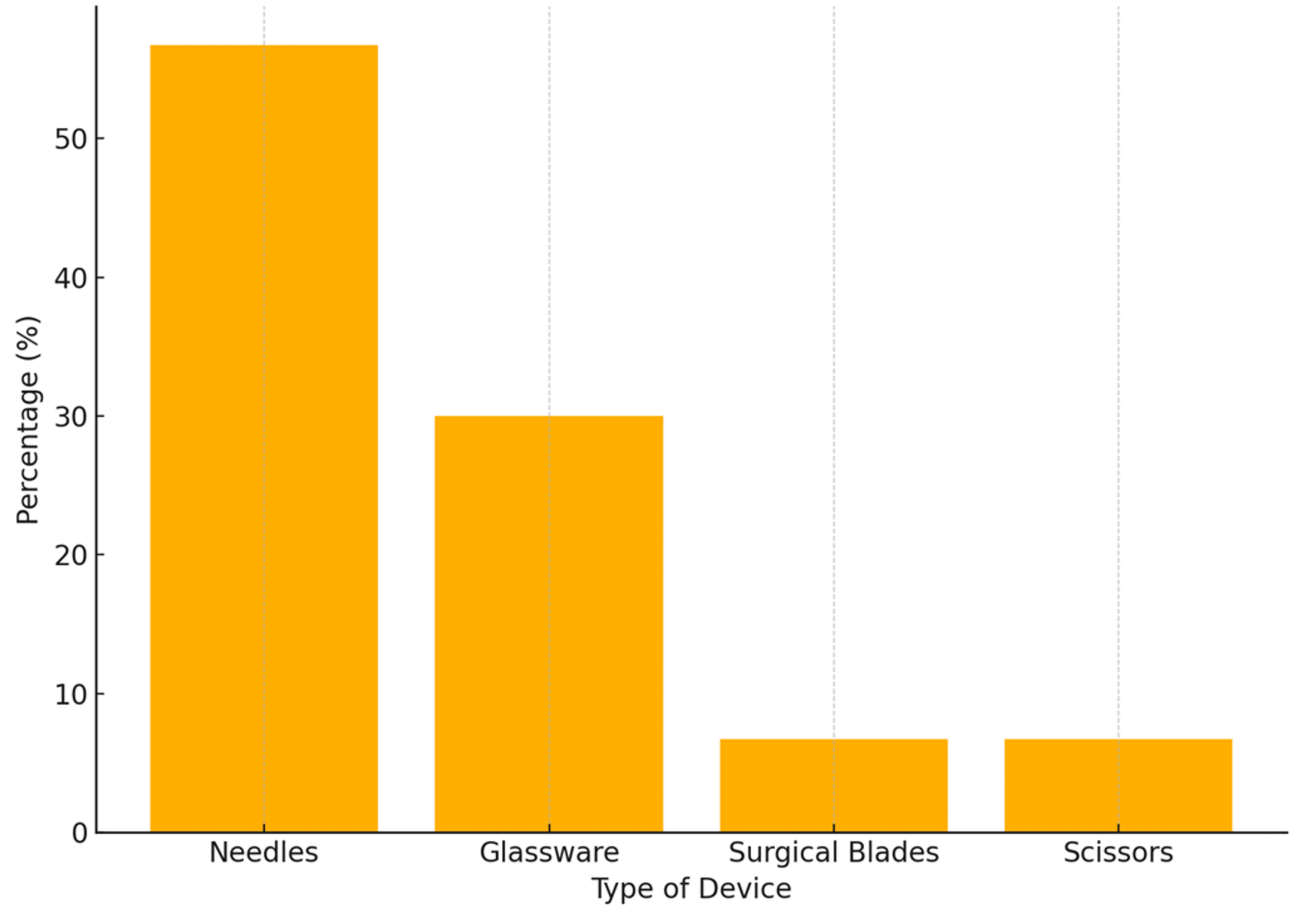
Type of device causing sharps injury

Among the nurses who reported experiencing sharps injuries, 14 of them (46.7%) had an injury with a moderate severity. Eleven (36.7%) of the affected nurses reported minor injuries, and three (10%) reported severe injuries. Needles accounted for 17 (56.7%) of the injuries caused by the devices, making them the most often implicated. This was followed by glassware, which caused nine (30%) of the injuries, while surgical blades and scissors were each responsible for two (6.7%) of the incidents (Figure 3). Sharps injuries arise most often throughout the preparation and usage of medical devices. Specifically, 10 (33.3%) of the injuries happened while preparing the device, and nine (30%) occurred during actual use. Recapping was the third most common activity associated with injury, reported in six (20%) of cases. Injuries during the disassembling and disposal of sharps accounted for two (6.7%) and three (10%), respectively. The most commonly cited factor of exposure was a busy clinical schedule, reported by 17 (56.7%) of the nurses. Other contributing factors included uncooperative patients (four, 13.3%), negligent behaviour (four, 13.3%), lack of assistance (two, 6.7%), failure to wear gloves (two, 6.7%), and tiredness (one, 3.3%). The frequency of nurses suffering injuries from sharp objects, as displayed in Figure 3, indicates that 8.84% of study participants reported experiencing at least one sharps injury incident.

Following exposure to sharps, 24 (80%) nurses reported washing the site of injury with running water alone, while only six (20%) of them used both running water and soap.

Regarding the reporting of incidents, 17 (56.7%) of nurses informed the unit nursing in-charge or team leader. Others (six, 20%) reported to the emergency room nurse, the infection control nurse (three, 10%), or to all of them (four, 13.3%). Regarding the incidence of non-reporting in previous exposures, a total of 18 (60%) injuries went unreported. The reasons for non-reporting included perceiving the injury as minor (six, 33.3%), lack of time (four, 22.2%), unawareness of proper guidelines (four, 22.2%), indifference to the need for reporting (two, 11.1%), and no visible evidence of injury (two, 11.1%). Post-exposure prophylaxis (PEP) was initiated promptly in more than half of the total affected nurses. Specifically, 18 (60%) of them received PEP within 24 hours of the incident, while two (6.7%) nurses received it after 24 hours. Among those who did receive prophylaxis, tetanus toxoid (TT) was the commonly administered intervention, provided to 19 (63.3%) of recipients. While one (3.3%) nurse received antiretroviral therapy (ART). The remaining 10, that is, 33.3% of affected nurses, received no prophylaxis.

There was a significant lack of follow-up antibody tests on exposure. Most nurses (63.3%) were not subjected to any kind of antibody test after the incident. On 11 (36.6) occasions, the nurses conducted an antibody test following a sharps injury. Such results reflect a loophole in the protocols of post-exposure monitoring. The affected nurses had limited refresher training after sharp injuries. Just a fifth of them were informed that they were trained on biomedical waste management after the incident. Fewer, only 16.7%, were given refresher training on post-exposure prophylaxis. This absence of follow-up education is an indication of the necessity of more systematic and continuous post-incident training programs that should reinforce safe practices and ensure preparedness (Table 2).

**Table 2:** Frequency distribution of occupational exposure to sharps injury among study participants working at a tertiary care center, Bihar (n=30)

Variables	Frequency	Percentage (%)
Frequency of exposure to sharps injury within one year		
Once	18	60
Up to 5 times	12	40
Time of recent exposure to a sharp injury		
Within 1 month	7	23.3
1-6 months	13	43.3
6 months to 1 year	10	33.3
Interval between recent and previous exposures		
Less than 1 month	2	6.7
1-6 months	8	26.7
6 months to 1 year	20	66.6
Contamination status of object (recent exposure)		
Contaminated with blood	7	23.3
Contaminated with other body fluids	5	16.6
Unknown status	18	60
Source of injury (recent exposure)		
Needles	17	56.7
Glassware	9	30.0
Surgical blades	2	6.7
Scissors	2	6.7
Activity that led to injury (recent exposure)		
While preparing	10	33.3
While using the device	9	30
While disassemble	2	6.7
While disposing	3	10
While recapping	6	20
Reason for exposure(recent exposure)		
Busy schedule	17	56.7
Uncooperative patients	4	`13.3
Tiredness	1	3.3
Failure to wear gloves	2	6.7
Lack of assistance	2	6.7
Negligent behavior of nurses	4	13.3
Severity of injury (recent exposure)		
Exposed with no injury	2	6.7
Mild	11	36.7
Moderate	14	46.7
Severe	3	10
Infectious status of source (recent exposure)		
Non-infectious status	15	50
HIV positive	1	3.3
HBS Ag positive	3	10
HCV positive	1	3.3
Unknown status	10	33.3
Action taken after exposure (recent exposure)		
Washed hands in running water	24	80
Washed hands in running water using soap	6	20
Time taken to report (recent exposure)		
Within 1 hour	19	63.3
After 1 hour and within 24 hours	7	23.3
After 24 hours	4	13.3
Contact of reporting(recent exposure)		
Infection control nurse	3	10
Emergency room nurse	6	20
Unit nursing in charge or team leader	17	56.7
All of them	4	13.3
Incidence of non-reporting in previous exposures		
No	12	40
Yes	18	60
Reason for non-reporting (if Yes)		
Lack of time to report	4	22.2
Unaware of guidelines	4	22.2
Only a minor injury	6	33.3
Not bothered to report	2	11.1
No visible evidence of injury	2	11.1
Time of receiving post-exposure prophylaxis after recent exposure		
Within 24 hours	18	60
After 24 hours	2	6.7
No post-exposure prophylaxis received	10	33.3
Type of post-exposure prophylaxis received		
Tetanus toxoid (TT)	19	63.33
Antiretroviral therapy (ART)	1	3.33
No post-exposure prophylaxis received	10	33.33
Time taken to test the antibody after recent exposure		
Antibody testing performed	11	36.6
No antibody testing performed	19	63.3
Refresher training on biomedical waste management was received after recent exposure.		
Received	6	20
Not received	24	80
Refresher training on post-exposure prophylaxis received after recent exposure		
Received	5	16.7
Not received	25	83.3

Association between sharps injury and baseline characteristics

The association between baseline features and sharp injury was ascertained using inferential statistics such as Fisher's exact test and the Chi-square test. In the case of variables with fewer than five cells, the exact test of Fisher was used. A multivariate analysis was then carried out to identify independent predictors of injuries caused by sharp objects. Age was found to have a statistically significant impact on the likelihood of injury (p < 0.05) after the possible confounding factors were factored in; however, the effects of gender and marital status were found to be less significant, which indicates the possibility of collinearity between the two variables. Meanwhile, no independent significant association was found between baseline characteristics such as educational status, unit of work, years of experience, Hepatitis B vaccination, biomedical waste management training, training on post-exposure prophylaxis, and sharps injury (p > 0.05) (Table 3).

**Table 3:** Association between baseline characteristics and sharps injury among nurses (n=339) Significant at p < 0.05 (shown with an asterisk *). Chi-square test was used for categories with cell counts ≥ 5, and Fisher’s exact test was used for categories with cell counts < 5.

Variables	Categories	Sharps injury (n (%))	No sharps injury (n (%))	p value
Age	20-24 years	9 (30)	20 (6.5)	0.002*
	25-29 years	17(56.7)	200(64.7)	
	30-34 years	4(13.3)	80 (24.9)	
Gender	Male	5 (16.7)	107 (34.6)	0.046*
	Female	25 (83.3)	202 (65.4)	
Marital status	Unmarried	24(80)	156 (50.5)	0.002*
	Married	6 (20)	153 (49.5)	
Educational status	General Nursing and Midwifery	1(3.3)	35(11.3)	0.316
	Post B.Sc. Nursing	3 (10)	23(7.4)	
	B.Sc. Nursing	26(86.7)	235 (76.1)	
	M.Sc. Nursing	0 (0)	16 (5.2)	
Unit of work	Emergency	3 (10)	23 (7.4)	0.103
	OPD	1(3.3)	49 (15.9)	
	Ward	10 (33.3)	120 (38.8)	
	ICU	8 (26.7)	66 (21.4)	
	Operating room	3 (10)	34 (11)	
	Labour room	4 (13.3)	13 (4.2)	
	Blood bank	0 (0)	2 (0.6)	
	Vaccination area	1 (3.3)	2 (0.6)	
Years of experience	Less than 1 year	12(40)	81(26.2)	0.070
	1-5 years	18(60)	176(57)	
	6-10 years	0(0)	48(15.5)	
	11-15 years	0(0)	3(1)	
	More than 15 years	0(0)	1(0.3)	
Hepatitis B vaccination	Completely vaccinated	17(56.7)	155(50.2)	0.538
	Partially vaccinated	5(16.7)	81(26.2)	
	Unvaccinated	8(26.7)	73(23.6)	
Training on biomedical waste management	Received	20(66.7)	249(80.6)	0.106
	Not received	10(33.3)	60(19.4)	
Training on post-exposure prophylaxis	Received	16(53.3)	211(68.3)	0.106
	Not received	14(46.7)	98(31.7)	

## Discussion

Hepatitis B virus (HBV), hepatitis C virus (HCV), and HIV are among the blood-borne illnesses that nurses frequently encounter, making sharps injuries a major occupational health hazard for healthcare workers. Needlesticks are the most prevalent profession-related sharps injury among workers, according to prior research [16-19], and the number of injuries reported in this study was caused by needle sticks. The study's results are similar to those of Nagao et al. (2007), who also documented injuries sustained after recapping the needle (14.7%) and disassembling a device (1.2%) [20]. Similarly, Clarke et al. (2002) found disposal of (32.5%) and recapping of needles (15.2%) were linked with sharps injuries [21]. These collective findings highlight the fact that procedural and behavioural factors, as opposed to institutional or geographical differences, are, for the most part, responsible for variations in injury rates amongst healthcare professionals.

Despite the lower prevalence across the board, the study found a number of worrisome trends. Vaccination rate against HBV was moderate, with only 50.7% nurses having complete immunization, 25.4% having partial immunization, and 23.9% having no immunization, which demonstrated the urgent need for special immunization activities [22]. Sharps injuries were most common in high-demand clinical areas such as general wards and intensive care units, and were often related to critical procedures, such as device preparation and use, and recapping of the sharps. Furthermore, although the majority of injuries were of low-to-moderate severity, a significant number were not reported or were not managed well. This indicates continuous lapses in the observance of safety practices, even with the availability of training programmes to train nurses in the institution [23].

Multivariate analysis showed that both age and gender were significant independent predictors of sharps injuries (p < 0.05). Although the significance of marital status no longer appeared after adjustment, the observed relationships between age, gender, and marital status indicated some degree of collinearity. Younger and mainly female nurses reported more injuries, suggesting that inexperience, heavy workload, and higher patient-handling responsibilities may contribute more to the risk of injury than might gender or their marital status alone. In the Indian sociocultural context, where the younger female nurses are more often in junior posts and do more direct patient-care work, these findings highlight the need for better supervision, ongoing safety training, and mentoring of early-career nurses to reduce the hazards of sharps.

Limitations

In order to estimate the prevalence of sharps injuries, this research relied on participant self-reported data; hence, it was unable to eliminate the risk of information bias, including social desirability and recall bias. Furthermore, by definition, a cross-sectional study is unable to identify any risk variables by establishing a cause-and-effect relationship. Future research could compare different healthcare professionals in various contexts.

Recommendations

In view of the results, some suggestions are offered to raise workplace safety generally and lower the incidence of sharps-related occurrences. These include implementing task management techniques, improving recurring training programs for safe sharps handling and biomedical waste management, and conducting routine monitoring to guarantee 100% hepatitis B vaccination coverage. When combined, these actions are intended to support the development of a culture of safety, responsibility, and readiness among nursing staff members who operate in clinical settings that carry a high level of risk.

## Conclusions

Needlestick injuries and other sharps-related injuries continue to be a serious workplace risk for healthcare workers, especially in institutions that see a lot of patients from all over the world. To minimize the risk, healthcare officials ought to focus on compulsory hepatitis B vaccination and periodic training on prevention of sharps injury and post-exposure prophylaxis, especially among nurses, as quality matters more than quantity. To create more accurate prevention strategies, future research ought to examine the risks of sharps injuries in various healthcare employees and in various clinical environments.

## Appendices

Questionnaire

Section - I: Baseline Characteristics (Socio-Demographic and Professional Information)

1. Age in years ☐ 20-24 ☐ 25-29 ☐ 30-34 ☐ 35-39

2. Gender ☐ Male ☐ Female

3. Marital Status ☐ Unmarried ☐ Married

4. Educational Status ☐ GNM ☐ Post B.Sc Nursing ☐ B.Sc Nursing ☐ M.Sc Nursing 

5. Working Unit ☐ Emergency room ☐ OPD ☐ Wards ☐ Intensive Care Unit ☐ Operating Room ☐ Labour Room ☐Blood Bank ☐ Vaccination Area

6. Years of Experience ☐ Less than 1 year ☐ 1-5 years ☐6-10 years ☐ 11-15 years ☐ More than 15 years

7. Hepatitis B Vaccination ☐ Completely Vaccinated ☐ Partially Vaccinated ☐ Unvaccinated

8. Training on Biomedical Waste Management ☐ Received ☐ Not received

9. Training on Post-Exposure Prophylaxis ☐ Received ☐ Not received

Section - II: Factors Associated With Sharps Injury

1. Frequency of exposure to sharps injury within one year ☐ Once ☐ Upto five times

2. Time of recent exposure to a sharp injury ☐ Within one month ☐ 1-6 months ☐ Within one year

3. Interval between recent and previous exposures ☐ Less than one month ☐ 1-6 months ☐ 6 months to 1 year

4. Contamination status of object (recent exposure) ☐ With blood ☐ With other body fluids ☐ Unknown status

5. Source of injury (recent exposure) ☐ Needles ☐ Glassware ☐ Surgical blades ☐ Scissors

6. Activity that led to injury (recent exposure) ☐While preparing the device ☐ While using the device ☐ While disassembling ☐ While recapping

7. Reason for exposure (recent exposure) ☐ Busy schedule ☐ Uncooperative patients ☐ Tiredness ☐ Failure to wear gloves ☐ Lack of assistance ☐ Negligent behavior of nurses

8. Severity of injury (recent exposure) ☐ Exposed with no injury ☐ Mild ☐ Moderate ☐ Severe

9. Infectious status of source (recent exposure) ☐ Non-infectious status ☐ HIV positive ☐ HBS Ag positive ☐ HCV positive ☐ Unknown status

10. Action taken after exposure (recent exposure) ☐ Washed hands in running water ☐ Washed hands in running water using soap

11. Time taken to report (recent exposure) ☐ Within one hour ☐ After one hour and within 24 hours ☐ After 24 hours

12. Contact of reporting(recent exposure) ☐ Infection control nurse ☐ Emergency room nurse ☐ Unit incharge ☐ All of them

13. Incidence of non-reporting in previous exposures ☐ No ☐ Yes

14. Reason for non-reporting (if Yes) ☐ Lack of time to report ☐ Unaware of guidelines ☐ Only a minor injury ☐ Not bothered to report ☐ No visible evidence of injury

15. Time of receiving post-exposure prophylaxis after recent exposure ☐ Within 24 hours ☐ After 24 hours ☐ No post-exposure prophylaxis received

16. Type of post-exposure prophylaxis received ☐ Tetanus toxoid (TT) ☐ Antiretroviral therapy (ART) ☐ No post-exposure prophylaxis received

17. Time taken to test the antibody after recent exposure ☐ Antibody testing performed ☐ No antibody testing performed

18. Refresher training on biomedical waste management was received after recent exposure ☐ Received ☐ Not received

19. Refresher training on post-exposure prophylaxis was received after recent exposure ☐ Received ☐ Not received
